# P-1191. Maternal Antibodies Against Parechovirus-A3 Before and During the Coronavirus Disease 2019 Pandemic

**DOI:** 10.1093/ofid/ofae631.1375

**Published:** 2025-01-29

**Authors:** Jun Tachikawa, Yuta Aizawa, Kanako Watanabe, Kazufumi Haino, Koji Nishijima, Akihiko Saitoh

**Affiliations:** Department of Pediatrics , Niigata University Graduate School of Medical and Dental Sciences, Niigata, Japan, Niigata, Niigata, Japan; Niigata University Graduate School of Medical and Dental Sciences, Niigata, Niigata, Japan; Niigata university, Niigata, Niigata, Japan; Department of Obstetrics and Gynecology, Niigata University Graduate School of Medical and Dental Sciences, Niigata, Japan, Niigata, Niigata, Japan; Department of Obstetrics and Gynecology, Niigata University Graduate School of Medical and Dental Sciences, Niigata, Japan, Niigata, Niigata, Japan; Niigata University, Niigata, Niigata, Japan

## Abstract

**Background:**

Parechovirus-A3 (PeV-A3) is an important emerging virus that causes severe disease in neonates and young infants. The coronavirus disease 2019 (COVID-19) pandemic greatly affected the incidence of infectious diseases, including PeV-A3 infection. Before the pandemic, we reported that maternal neutralizing antibody response against PeV-A3 was important in protecting neonates and young infants from PeV-A3 and that neutralizing antibody titers (NATs) were higher in younger mothers (*Emerg Infect Dis*. 2015;21:1966-1972). Because the impact of the COVID-19 pandemic on maternal NATs against PeV-A3 is unclear, we investigated NATs against PeV-A3 during the COVID-19 pandemic and compared our findings with previous data.

**Figure:**
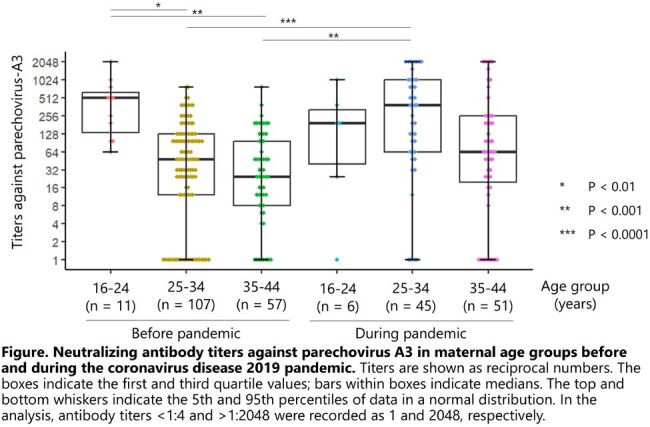

**Methods:**

Cord blood (CB) samples for healthy newborns born at full-term were collected from June through December 2023 at Niigata University Hospital in Niigata, Japan. Maternal clinical data were collected, and NATs measured using LLC-MK2 cells were compared with the pre-pandemic data, classified by age group, ie, 16–24 years, 25–34 years, and 35–44 years.

**Figure:**
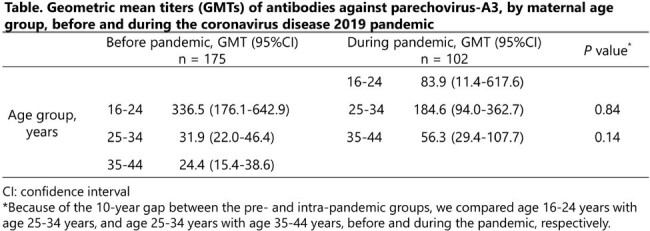

**Results:**

In total, 102 CB samples were collected. Median maternal age was 34.5 years (range: 30–37 years), and the geometric mean titer (GMT) of antibodies against PeV-A3 was 98.4 (95% CI: 61.7–156.8). The GMT values for age 25–34 years during the pandemic (184.6, n = 45) and age 16–24 years before the pandemic (336.5, n = 11) did not significantly differ (P = 0.84). Similarly, the GMT for age 35–44 years during the pandemic (56.3, n = 51) did not significantly differ from that for the matching age group, 25–34 years, before the pandemic (31.9, n = 107) (P = 0.14). Notably, the GMT was significantly higher for age 25–34 years during the pandemic than for age 25–34 years and age 35–44 years before the pandemic (P < 0.001), which is consistent with our finding that the pre-pandemic age group 16–24 years had the highest GMT.

**Conclusion:**

Maternal NATs against PeV-A3 in CB before and during the COVID-19 pandemic were similar, which suggests that humoral immunity to PeV-A3 was not substantially altered even after 3 years of limited PeV-A3 infections during the COVID-19 pandemic.

**Disclosures:**

**All Authors**: No reported disclosures

